# A single session of exercise reduces blood pressure reactivity to stress: a systematic review and meta-analysis

**DOI:** 10.1038/s41598-022-15786-3

**Published:** 2022-07-12

**Authors:** Igor M. Mariano, Ana Luiza Amaral, Paula A. B. Ribeiro, Guilherme M. Puga

**Affiliations:** 1 Laboratory of Cardiorespiratory and Metabolic Physiology, Physical Education Department, Federal University of Uberlândia, Rua Benjamin Constant, 1286, Neighborhood: Aparecida, Uberlândia, MG 38400-678 Brazil; 2 @CoeurLab Research Unit, Research Center of University of Montreal Hospital Centre, Montreal, QC H2X 0A9 Canada; 3 Centre de Médecine Comportemental de Montréal, CIUSSS- NIM, Montreal, QC H4J 1C5 Canada

**Keywords:** Lifestyle modification, Cardiology

## Abstract

Stressful situations are common in everyday life and disturb homeostasis. So, an exercise session is a strategy to mitigate blood pressure (BP) peaks in response to stress (i.e., BP reactivity), decreasing the cardiovascular risk. This is a systematic review and meta-analysis that aims to verify the effects of a single session of physical exercises on BP reactivity to stress in adults. The searches were performed in digital databases (MEDLINE, LILACS, EMBASE, SPORTDiscus, and PsycInfo) and 29 studies were included, totaling 795 individuals (quantitative analysis: k = 25, n = 659). As for exercise characteristics, 21 of the 29 studies focused on aerobic exercises, and 23 studies focused on low to moderate intensities. As for the stress tests, we have them in the following order from the most to the least frequent: stroop color and word test, cold pressor test, arithmetic test, public speaking, handgrip, trier social stress test, and study task. Favorable metanalytic results (standardized mean differences through random-effects approach) for the exercises were found, with attenuated reactivity in systolic BP (pooled effect size = − 0.38 [− 0.49; − 0.27], representing average reductions of 3.7 ± 3.8 mmHg), diastolic BP (pooled effect size = − 0.51 [− 0.70; − 0.33], representing average reductions of 2.9 ± 3.7 mmHg), and mean BP (pooled effect size = − 0.51 [− 0.72; − 0.31], representing average reductions of 4.1 ± 3.3 mmHg). So, acute physical exercise lowers systolic, diastolic, and mean blood pressure reactivity in response to stressor tasks. However, given the small magnitude of effects, the clinical relevance of this result must be interpreted with caution and be better explored.

## Introduction

Stressful situations are common in modern life and can cause transient alterations in autonomic, catecholaminergic, and neural networks in response to it^1–3^. Although these alterations are expected to prepare the body for the challenge, prolonged, frequent, or exaggerated responses to stress can be indicative of future cardiovascular risk^2^. In this way, simple laboratory stress tests that disturb the homeostasis in a controlled manner were previously associated with the development of future cardiovascular events, depression, and decreased telomere length^4^. These tests involve different types of stressors, such as physical (e.g., cold), mental (e.g., arithmetic task), or a mix of both^5^. Besides, one of the simplest and most frequent ways to assess stress reactivity responses is based on changes in blood pressure (BP) (i.e., hypertensive peaks)^5^.

In a broad context, high BP is one of the main preventable factors associated with premature death globally^6^ and is associated with the risk of cardiovascular events, strokes, and kidney disease^7^. In this context, one of BP's control strategies is to perform physical exercises. Evidence shows that even after a single exercise session, BP can be below baseline levels at rest^8^ but its influence on BP reactivity to stressful situations is still poorly understood. Despite that, it has already been suggested that cardiovascular responses to stress are better indicators of left ventricular mass^9^ and the development of hypertension^10,11^ than resting BP, reiterating the importance of studying these responses.

In 2006, a meta-analysis by Hamer and collaborators^12^ evaluated the acute effects of aerobic exercise on BP reactivity to several laboratorial stress tests (i.e. stroop color and word test, arithmetic test, cold pressor test, and study task) and found favorable results with attenuated hypertensive peaks in adults (effect size between 0.38 and 0.40). However, in addition to new studies being produced since then, responses to non-aerobic exercise are still unclear. Thus, the aim of the present systematic review and meta-analysis is to verify the acute effects of physical exercise on stress-related BP reactivity in adults. The hypothesis is that the exercise will be able to mitigate stress reactivity, with a similar magnitude to those demonstrated in isolated aerobic exercises^12^.

## Methods

This systematic review and meta-analysis followed PRISMA guidelines^13,14^, had its protocol published (available at: 10.17504/protocols.io.bhw3j7gn)^15^, and was registered on PROSPERO (CRD42020194353).

### Eligibility criteria

Studies with the following characteristics were eligible: (1) population: human, both sexes, adults (i.e. > 18 years), regardless of health or training status; (2) intervention: a session of physical exercise; (3) control: a session without exercise; (4) outcome of interest: BP reactivity under stress (peak BP during a stress test or BP variation from basal levels); (5) languages: English, Portuguese or Spanish; (6) study designs: randomized clinical trials or crossovers; (7) publication dates: no time limit; (8) other characteristics: in studies with more than two intervention arms, only comparisons with the control group were considered, dividing the control sample proportionately to avoid sample duplication in the final analysis.

### Search strategy

**Figure 1:**
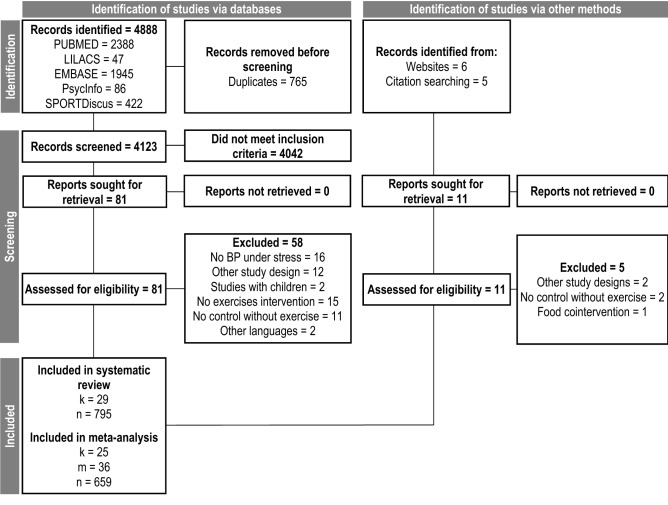
Flow diagram. k, number of studies; n, pooled sample size; BP, blood pressure.

The searches were performed on April 26th/2022, in digital databases (MEDLINE, LILACS, EMBASE, SPORTDiscus, and PsycInfo). Also, in the reference lists of the included studies, and through manual search on other websites (“https://core.ac.uk/” and “https://scholar.google.com/”). The flow diagram is shown in Fig. 1, and the list of studies excluded from full-text screening are available in Supplementary Data S1. The search was organized into the following categories of terms: exercise intervention, BP, and stressors. Parentheses and intersection boolean operators (i.e. “AND”) were used to separate the categories, and union operators (i.e. “OR”) were used to separate the terms of each category. In this way, these terms were searched in title, abstract, and keywords indexed in the aforementioned databases in the following format:

*(Exercise OR “Exercise Therapy” OR “Physical activity” OR “Physical training” OR Aerobic OR Cycling OR Bicycle OR Treadmill OR “Cycle ergometer” OR Cyclergometer OR “Cycle-ergometer” OR Swimming OR Swim OR Running OR Run OR “Hand grip” OR “Hand-grip” OR Walking OR Walk OR "Weight training” OR "Weight-training” OR “Weight exercise” OR “Weight-exercise” OR “Resistance exercise” OR “Resistance training” OR Strength OR Pilates OR Yoga OR Ioga OR Taichi OR “Tai chi” OR “Tai-chi” OR Isometric OR Hiit OR Hit OR Siit OR Sit OR “High intensity” OR “Moderate intensity” OR ”Low intensity” OR “Combined training” OR “Combined exercise” OR “Concurrent training” OR “Concurrent exercise”) AND (“Arterial pressure” OR "Blood pressure" OR Diastolic OR Systolic) AND ("Reactivity" OR "Cold pressor" OR "Stroop" OR "Stress test" OR Psychosocial OR “Psychosocial test” OR “Psychosocial stress” OR “Psychosocial task” OR “Stress task” OR “math task” OR “Speech task” OR Speech OR Math OR Arithmetic OR “Arithmetic test” OR “Arithmetic task”).*

### Screening and data extraction process

During the process of screening (title and abstract, and full-text), data extraction, and risk of bias assessment, the studies were evaluated in duplicate by independent reviewers. After checking the responses, the reviewer’s disagreements were resolved by consensus or by a third reviewer when necessary. The reviewer’s agreement was estimated from Cohen's kappa in both full-text screening (κ = 0.671; *p* < 0.001; 13 disagreements were resolved by a third reviewer) and risk of bias assessment (κ = 0.867; *p* < 0.001).

Before the data extraction phase, one of the reviewers standardized codes for all studies included in the following analyses. Thus, each reviewer independently filled an electronic datasheet detailing the characteristics of the studies and the data was compared to assess agreement and identify errors. This datasheet included: identification code, author last name, publication year, language, study design, sample sizes, health and fitness status, age, sex, hypertension status, other comorbidities, exercise intensity, exercise volume (measured in minutes), exercise mode (aerobic, resistance, combined or yoga), stressor test, BP measure device/technique, and BP reactivity measures (sample sizes, mean and standard deviation. If other types of measures were reported, the mean and standard deviation were requested from the authors, and in case of null or negative answers, the results were transformed (when possible). When there was not sufficient data for meta-analysis, the authors were contacted to request further information. Studies in which the data are presented without numerical description, it was extracted through a web-based software (https://automeris.io/WebPlotDigitizer).

### Statistical analysis

Pooled estimates were calculated using standardized mean differences (SMD) with confidence intervals (95% CI), using “R” programming language through the packages "meta"^16^ and "metafor"^17^. For the pooled effect, were considered the values of BP reactivity under stress (peak BP during a stress test or BP variation from basal levels) after an exercise session and after a control session without exercise, as a comparator. In studies with multiple stressors, we used the mean and pooled dispersion between the stressors. The heterogeneity was measured by I^2^ and Kendall's tau using the Hunter Smith method for heterogeneity variance estimators^18,19^. Due to the different characteristics of interventions, population, and stress tests, we selected a random-effects approach to summarize the metanalytic results.

The sensitivity analysis was done through the search for outliers and influential points using externally standardized residuals (values farther than 1.96 standard deviations in the standardized residuals graph), difference in fits (identifying values above 1 or below − 1), covariance ratio (identifying values below 1) and Cook’s distance methods (identifying values far above the other studies). In addition, we visually evaluated the overlap of confidence intervals in the forest plot, and studies without overlap would be considered outliers. In addition, subgroup analyses by type of stressor, the number of stressors, participants' sex, exercise mode, and studies design were made. The individual study assessment of the risk of bias was conducted through “Risk of Bias 2.0” method from the Cochrane collaboration^20^ and its graphical visualization by the “R” package "robvis"^21^. Publication bias analysis was carried out through Egger’s regression^22^ and trim and fill funnel plots^23^. Quality of evidence was accessed throught Grading of Recommendations Assessment, Development and Evaluation (GRADE) approach^24^.

## Results

### Studies characteristics

Studies included 388 women, 387 men, and 20 individuals in which sex was not disclosed. In addition, of the 29 studies^25–53^, only 4 (14%) included hypertensive patients^31,39,44,53^, 22 (76%) had a mean age of less than 30 years^25–30,32–34,37,40–43,46,50–52,54–56^, 4 (14%) were from 30 to 40 years old^35,38,47,48^, and only 4 (14%) were over 40 years old^31,39,44,53^. As for stress tests, we have as the most frequent the stroop color and word test (13 studies)^29,31–33,36–38,43,44,46–49^, followed by cold pressor^32,34,35,37,39,41,43,50–53^ (11 studies), arithmetic test^25,27,29,30,39,40,42,45,48^ (9 studies), public speaking (3 studies)^29,32,38^, handgrip (2 studies)^36,48^, and Trier Social Stress Test^28^ and Study task^26^ (1 study each). As for the time interval between the exercise session and the stressor task, only 2 studies (7%) performed more than 60 min later^42,44^, 7 studies (24%) performed between 31 and 60 min later^29,32,35,40,49,53,54^ and 23 studies (79%) performed in up to 30 min later^25–27,30,31,33,34,37–41,43,45–48,50–52,57^.

As for exercise characteristics, 2 studies included intervention with Yoga (7%)^30,34^, 4 (14%) with resistance exercises^41,42,51,52^ and 2 (7%) with combined exercises^35,53^, all the others focused on aerobic exercises (21 studies, 72%)^25–29,31–33,36–40,43–50,53^. Furthermore, the exercise sessions lasted between 10 and 120 min (average of 30–60 min). As for intensity, 1 study used self-selection^34^, 5 used high intensity^29,42,49,50,52^ and all others used low to moderate intensity (50–85% of the individual maximum; e.g. heart rate max, 1RM, VO_2max_)^25–28,30–33,35–41,43–48,51,53^.

Regarding experimental designs, 5 (17%) studies used a randomized clinical trial approach^25,27,29,32,46^, and 24 (83%) adopted a crossover design^26,28,30,31,33–45,47–53^. As the main results, 13 (45%) studies demonstrated improvements in systolic blood pressure (SBP)^25,31,32,35,37,38,40,44,47,49,51–53^, 14 (48%) in diastolic blood pressure (DBP)^25,31,32,35,37–41,44,46,47,51,52^, and 8 (out of 12; 67%) in mean blood pressure (MBP)^29,31,37,38,46,47,51,52^. The others (12; 41%) had null results since no study has shown harmful BP reactivity effects of exercise^26–28,30,33,34,36,42,43,45,48,50^. Besides that, four studies did not present data dispersion measures to be included in the meta-analysis^25–28^. The general characteristics of all studies are shown in Table 1.

**Table 1:** Studies characteristics.

Study	Population	Stress test	Exercise	Reactivity results
^29^*	NT, 23 women + 17 men, 22 years, athletes, Rest MBP: 89	Arithmetic + Stroop color + Public speech	Aerobic (Maximum incremental test)	↓MBP
^30^	NT, 11 women + 13 men, 22 years, Rest BP: 108/60	Arithmetic	Yoga (30 min)	↔ SBP ↔ DBP
^31^	Borderline HT, 8 participants, 41 years, Rest BP: 137/85	Stroop color	Aerobic (treadmill, 60 min, 60% VO_2max_)	↓SBP ↓DBP ↓MBP
^32^*	NT, 24 men, 22 years, Rest BP:122/72	Cold pressor + Stroop color + Public speech	Aerobic (60 min or 120 min, 55% VO_2max_)	Cold pressor: ↓SBP ↓DBP Other tests: ↔ SBP ↔ DBP
^33^	NT, 30 men, 21 years, Rest BP:123/68	Stroop color	Aerobic (20 min, 75–85% HR_reserve_)	↔ SBP ↔ DBP
^34^	NT, 9 women, 25 years, Rest BP: 119/57	Cold pressor	Yoga or Aerobic (20 min, auto select intensity)	↔ SBP ↔ DBP
^35^	NT, 10 women + 10 men, 33 years, Rest BP: 114/77	Cold pressor	Combined (30 min, 75–85% HR_max_ and 50% 1RM)	↓SBP ↓DBP
^36^	NT, 7 men, 23 years, Rest BP: 104/66	Hand grip + Stroop color	Aerobic (120 min, 50% VO_2max_)	↔ SBP ↔ DBP
^37^	NT, 12 men, 23 years, Rest BP: 114/67	Cold pressor + Stroop color	Aerobic (treadmill, 30 min, 60% VO_2max_)	Stroop Color: ↓SBP ↓DBP ↓MBP Cold pressor: ↔ SBP ↔ DBP ↔ MBP
^38^	NT, 48 women, 25–40 years, Rest BP: 109/63	Stroop color + Public speech	Aerobic (40 min, 70% HR_reserve_)	↓SBP ↓DBP ↓MBP
^39^	NT + HT, 18 women + 14 men, 47–51 years, Rest BP: 128/88	Arithmetic + Cold pressor	Aerobic (20 min, 60–70% HR_max_)	↔ SBP ↓DBP ↔ MBP
^40^	NT, 42 women + 48 men, 23 years, Rest BP: 116/69	Arithmetic	Aerobic (30 min, 50–55% VO_2max_ or 75–80% VO_2max_)	Both intensities: ↓SBP ↓DBP
^41^	NT, 6 women + 9 men, 26 years, Rest BP: 116/70	Cold pressor	Resistance (30 min, 40–60% 1RM)	↔ SBP ↓DBP
^42^	NT, 18 men, 20 years, Rest BP: 126/58	Arithmetic	Resistance (Eccentric movement, 45 min, 120% 1RM)	↔ SBP ↔ DBP
^43^	NT, 24 women (11 smokers), 21 years, Rest BP: 118/73	Cold pressor + Stroop color	Aerobic (30 min, 50% VO_2peak_)	↔ SBP ↔ DBP ↔ MBP
^44^	NT + HT, 12 women + 18 men, 41 years, Rest BP: NT: 120/74, HT: 144/94	Stroop color	Aerobic (53 min, 50% VO_2peak_)	↓SBP ↓DBP
^45^	NT, 11 men, 25 years, Rest BP: 123/70	Arithmetic	Aerobic (30 min, 70% HR_max_)	↔ SBP ↔ DBP ↔ MBP
^46^*	NT, 80 women, 18 years, Rest BP: 107/58	Stroop color	Aerobic (10 min or 25 min or 40 min, 70% HR_reserve_)	↔ SBP ↓DBP ↓MBP
^47^	NT, 12 participants, 31 years, Rest BP: 118/62	Stroop color	Aerobic (30 min at 50% VO_2max_ or 60 min at 80% VO_2max_)	50%: ↔ SBP ↓DBP ↓MBP 80%: ↓SBP ↓DBP ↓MBP
^48^	NT, 9 men, 32 years, Rest BP: 119/76	Hand grip + Stroop color + Arithmetic	Aerobic (30 min, 60% VO_2max_)	↔ SBP ↔ DBP
^49^	NT, 22 women + 4 men, 29 years, Rest BP: 116/68	Stroop color	Aerobic (Maximum incremental test)	↓SBP ↔ DBP ↔ MBP
^50^	NT, 22 men, 23 years, Rest BP: 124/75	Cold pressor	Aerobic (30 min at 50–60 HR_reserve_ or 20 min interval (4 × 3 min/2 min) at 80–90% HR_reserve_)	↔ SBP ↔ DBP
^51^	NT, 40 men, 26 years, Rest BP: 121/77	Cold pressor	Resistance (30 min or 50 min at 70% 1RM)	30 min: ↔ SBP ↔ DBP ↔ MBP 50 min: ↓SBP ↓DBP ↓MBP
^52^	NT, 13 men, 23 years, Rest BP: 115/68	Cold pressor	Aerobic (30 min at 55–60% HR_reserve_) or Resistance (20 min at 80–90* HR_reserve_)	Aerobic: ↓SBP ↔ DBP ↔ MBP Resistance: ↓SBP ↓DBP ↓MBP
^53^	HT, 15 women, 54 years, Rest BP: 119/75	Cold pressor	Combined (60 min at 75% of 8RM and walking at 14 of 20 from perceived exertion scale)	↓SBP ↔ DBP
**Included only in qualitative analysis**				
^25^*	NT, 15 men, 21 years, Rest BP: 128/68	Arithmetic	Aerobic (Cycle, 20 min at 25 or 100 watts)	25 watts: ↔ SBP ↔ DBP 100 watts: ↓SBP ↓DBP
^26^	NT, 18 women, undergraduate, Rest SBP: 112	40 min of study	Aerobic (40 min at 60–80% HR_max_)	↔ SBP ↔ DBP
^27^*	NT, 40 women + 40 men, 21 years, Rest BP: 115/70	Arithmetic	Aerobic (20 min at moderate intensity)	↔ SBP ↔ DBP
^28^	NT, 10 women + 13 men, 24 years, Rest SBP: 111	Trier Social Stress Test	Aerobic (30 min, 70% VO_2peak_)	↔ SBP

The age refers to the average. SBP: systolic blood pressure; DBP: diastolic blood pressure; MBP: mean blood pressure; HR: heart rate; HT: hypertensives; NT: normotensives; *: randomized clinical trials, the other studies are cross over designs.

### Meta-analysis results

**Figure 2:**
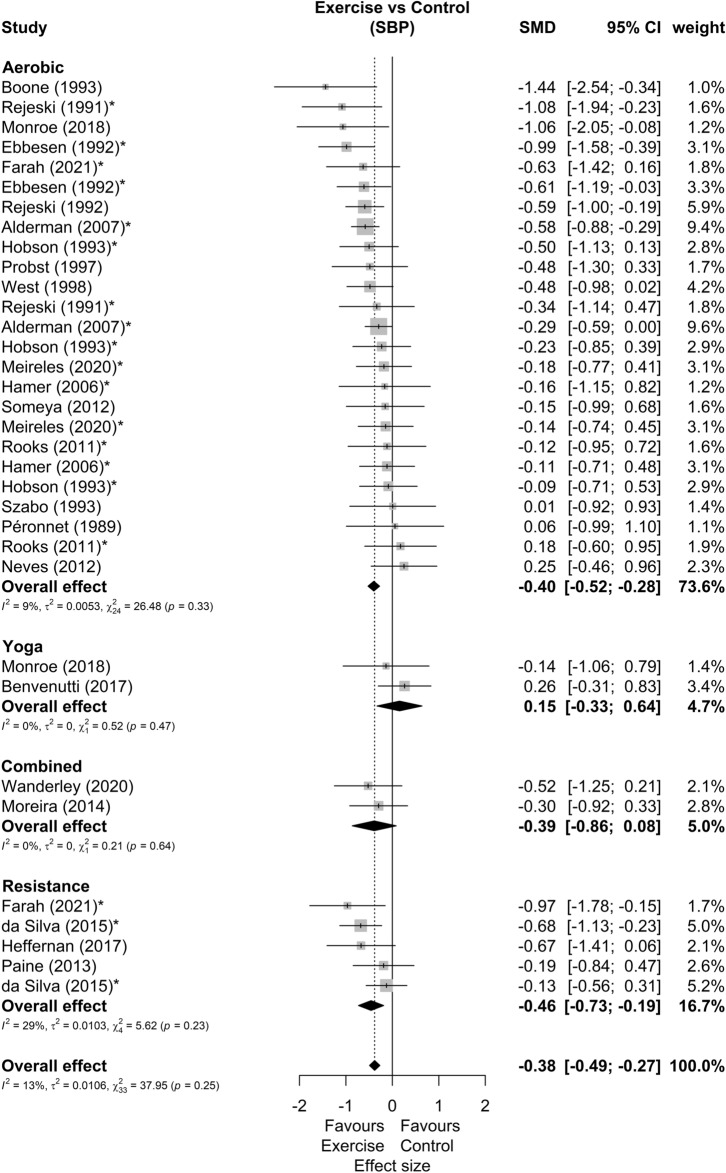
Systolic blood pressure reactivity forest plot. SMD, standardized mean difference; SBP, systolic blood pressure; CI, confidence interval; *, studies with multiple comparisons.

**Figure 3:**
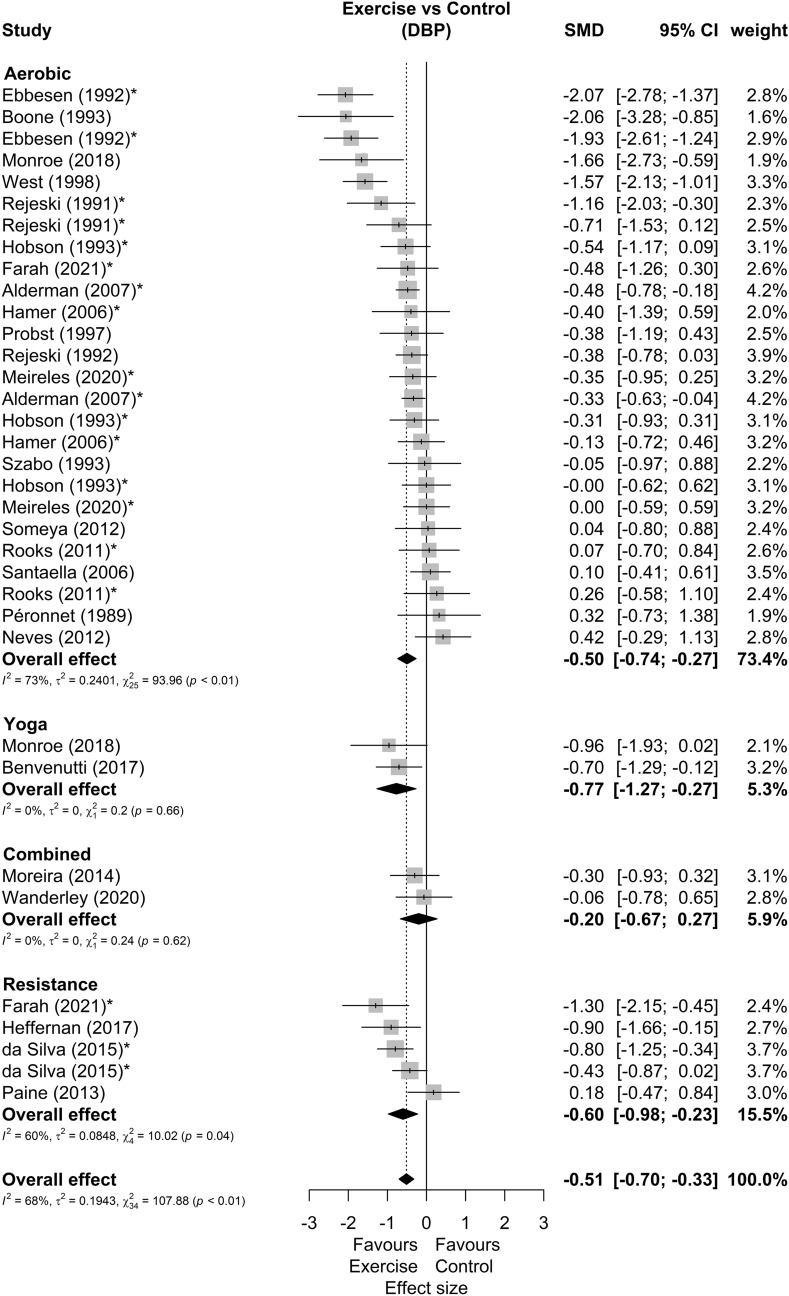
Diastolic blood pressure reactivity forest plot. SMD, standardized mean difference; DBP, diastolic blood pressure; CI, confidence interval; *, studies with multiple comparisons.

**Figure 4:**
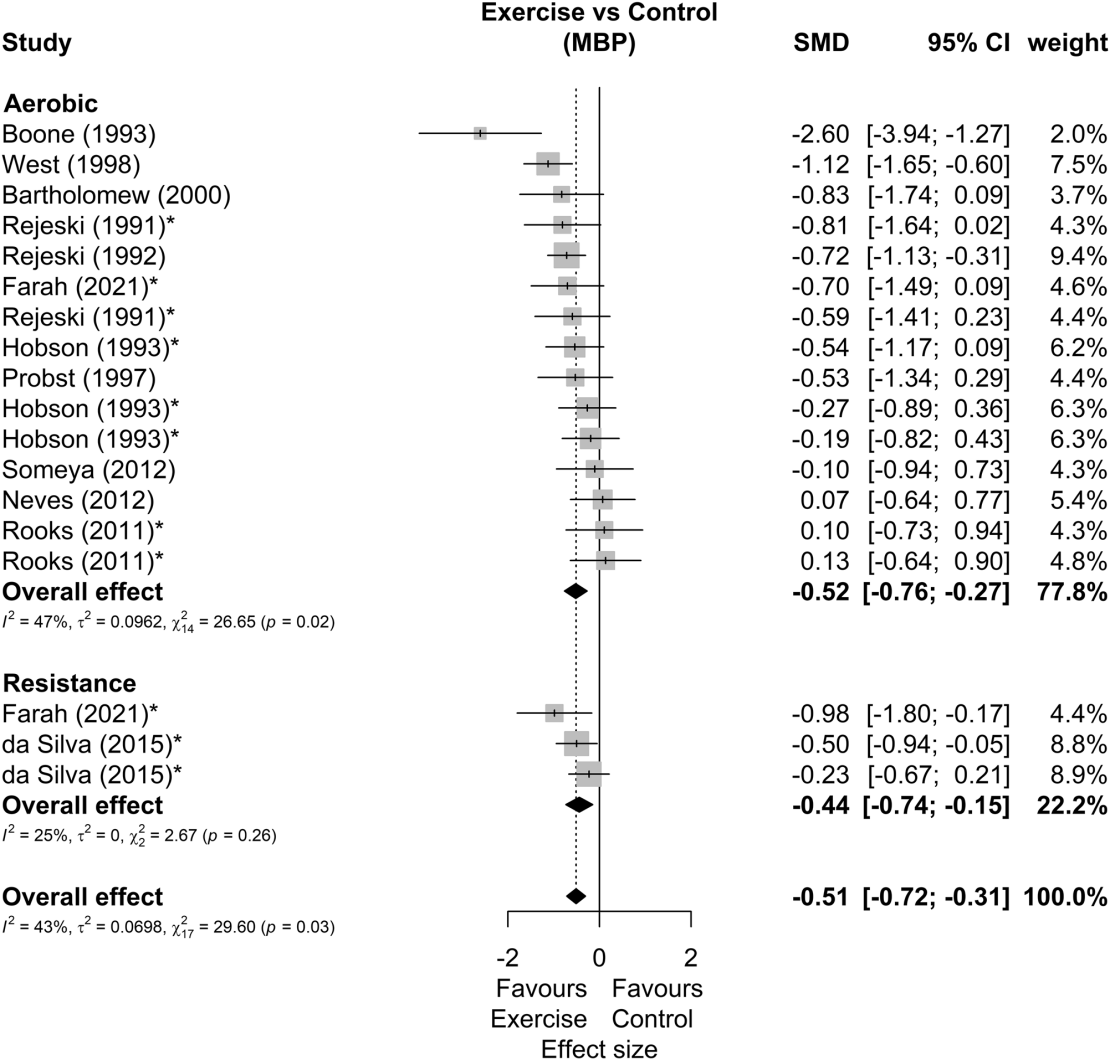
Mean blood pressure reactivity forest plot. SMD, standardized mean difference; MBP, mean blood pressure; CI, confidence interval; *, studies with multiple comparisons.

Among 25 studies included in meta-analysis^29–53^, 9 presented multiple possible comparisons according to the exercise mode^34,52^, exercise volume^32,46,51^, exercise intensity^47,50^, parents smoking habit^33^, or participants smoking habit^43^. Besides that, 23 studies demonstrate results for SBP (34 comparisons), 24 for DBP (35 comparisons) and 12 for MBP (18 comparisons), as shown in Table 1. The forest plots of SBP, DBP and MBP reactivity are present in Figs. 2, 3 and 4, respectively. We found small but favorable results to exercise in both SBP (SMD = − 0.38 [− 0.49; − 0.27], representing mean reductions of 3.7 ± 3.8 mmHg), DBP (SMD = − 0.51 [− 0.70; − 0.33], representing mean reductions of 2.9 ± 3.7 mmHg) and MBP reactivity (SMD = − 0.51 [− 0.72; − 0.31], representing mean reductions of 4.1 ± 3.3 mmHg). We also highlight that 20 (80%) of the studies were carried out in healthy non-athlete individuals aged up to 40 years ^29,30,32,34–37,40–43,46–52,55,58^. Thus, by isolating this population as an sensitivy analysis, we maintain the results for SBP (SMD = − 0.36 [− 0.48; − 0.25]), DBP (SMD = − 0.48 [− 0.67; − 0.30]), and MBP (SMD = − 0.41 [− 0.57; − 0.25]).

Other sensitivity analyses showed that 4 studies^31,32,39,49^ can be outliers and/or influential points in DBP and 1 study^31^ in MBP reactivity. New analysis disregarding these studies showed a DBP effect size of − 0.37 [− 0.50; − 0.24] and a MBP effect size of − 0.48 [− 0.65; − 0.31]. Subgroup analyses were performed in SBP and DBP, but none of these analyses reported significant differences between subgroups, either comparing: study design, participants sex, exercise mode, stress type or number of stressors. The summary of these analyses can be seen in Table 2. An additional analysis comparing the stressors showed no effects differences (SBP *p* = 0.81; DBP *p* = 0.47) between the cold pressor test (SBP SMD = − 0.42 [− 0.62; − 0.23]; DBP SMD = − 0.56 [− 0.80; − 0.32]), arithmetic test (SBP SMD = − 0.36 [− 0.54; − 0.17]; DBP SMD = − 0.36 [− 0.56; − 0.17]), stroop color and words test (SBP SMD = − 0.36 [− 0.65; − 0.06]; DBP SMD = − 0.35 [− 0.68; − 0.03]) or other tests (SBP SMD = − 0.51 [− 0.78; − 0.24]; DBP SMD = − 0.68 [− 1.24; − 0.13]).

**Table 2:** Summary of subgroup analysis for blood pressure responsiveness.

Subgroup variables	Effect size					Subgroup differences *p*	Heterogeneity		
	SMD	95% CI	Weight (%)	k	m		I^2^ (%)	τ^2^	Q
**SBP**									
**Sex**									
Men	− 0.38	[− 0.55; − 0.21]*	40.6	10	15	0.51	2	0.000	14.33
Women	− 0.48	[− 0.78; − 0.18]*	23.5	5	9		50	0.088	16.04*
Both	− 0.36	[− 0.54; − 0.18]*	31.1	6	7		22	0.005	7.72
Undefined	− 0.76	[− 1.27; − 0.24]*	4.8	2	3		0	0.000	1.80
**Exercise mode**									
Yoga	0.15	[− 0.33;0.64]	4.7	2	2	0.17	0	0.000	0.52
Aerobic	− 0.40	[− 0.52;− 0.28]*	73.6	15	25		9	0.005	26.48
Combined	− 0.39	[− 0.86; 0.08]	4.7	2	2		0	0.000	0.21
Resistance	− 0.46	[− 0.73;− 0.19]*	16.7	4	5		29	0.010	5.62
**Study design**									
RCT	− 0.49	[− 0.77; − 0.22]*	14.8	2	5	0.39	22	0.002	5.1
Cross over	− 0.36	[− 0.48; − 0.24]*	85.2	21	29		13	0.010	32.15
**Stressor type**									
Mental	− 0.40	[− 0.60; − 0.20]*	50	10	15	0.98	46	0.053	25.88*
Physical	− 0.42	[− 0.62; − 0.23]*	30.7	7	11		0	0.000	9.16
Both	− 0.42	[− 0.68; − 0.17]*	19.3	6	8		16	0.005	8.34
**Number of stressors**									
Multiple	− 0.51	[− 0.78; − 0.24]*	24.8	7	9	0.39	42	0.056	13.87
Unique	− 0.38	[− 0.50; − 0.26]*	75.2	16	25		11	0.006	26.86
SBP Overall	− 0.42	[− 0.54; − 0.30]*	100	23	34		24	0.025	43.38
**DBP**									
**Sex**									
Men	− 0.54	[− 0.87; − 0.20]*	41.7	10	15	0.07	74	0.302	55.55*
Women	− 0.32	[− 0.59; − 0.05]*	24.9	5	9		36	0.044	12.51
Both	− 0.47	[− 0.79; − 0.15]*	27.0	7	8		76	0.137	28.7*
Undefined	− 1.16	[− 1.72; − 0.59]*	6.4	2	3		39	0.022	3.29
**Exercise mode**									
Yoga	− 0.77	[− 1.27; − 0.27]*	5.3	2	2	0.40	0	0.000	0.24
Aerobic	− 0.50	[− 0.74; − 0.27]*	73.4	16	26		73	0.240	93.96*
Combined	− 0.20	[− 0.67; 0.27]	5.9	2	2		0	0.000	0.24
Resistance	− 0.60	[− 0.98; − 0.23]*	15.5	4	5		60	0.085	10.02
**Study design**									
RCT	− 0.96	[− 1.69; − 0.22]*	15.0	2	5	0.18	88	0.596	32.13*
Cross over	− 0.43	[− 0.61; − 0.26]*	85.0	22	30		57	0.113	67.21*
**Stressor type**									
Mental only	− 0.33	[− 0.51; − 0.14]*	48.1	11	16	0.22	44	0.050	26.89*
Physical only	− 0.56	[− 0.80; − 0.32]*	31.2	7	11		39	0.051	16.29
Both	− 0.72	[− 1.38; − 0.05]*	20.7	6	8		86	0.746	48.32*
**Number of stressors**									
Multiple	− 0.68	[− 1.24; − 0.13]*	24.5	7	9	0.38	85	0.573	53.5*
Unique	− 0.42	[− 0.58; − 0.26]*	75.5	17	26		46	0.068	46.21*
DBP overall	− 0.51	[− 0.70; − 0.33]*	100	24	35		68	0.194	107.88*

SBP, systolic blood pressure; DBP, diastolic blood pressure; SMD, effect size by standardized mean differences; CI, confidence interval; k, number of studies; m: number of comparisons; I^2^, Higgins e Thompson I^2^; Q, Cochran’s Q; τ^2^, Kendall’s τ^2^; *p < 0.05.

### Bias and quality of evidence assessment

**Figure 5:**
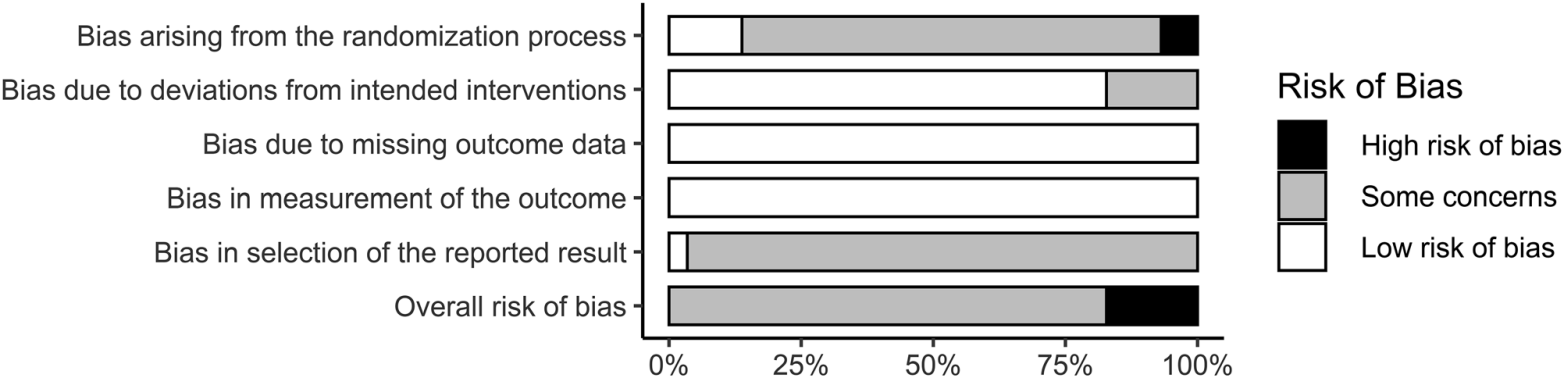
Risk of bias summary (k = 29).

**Figure 6:**
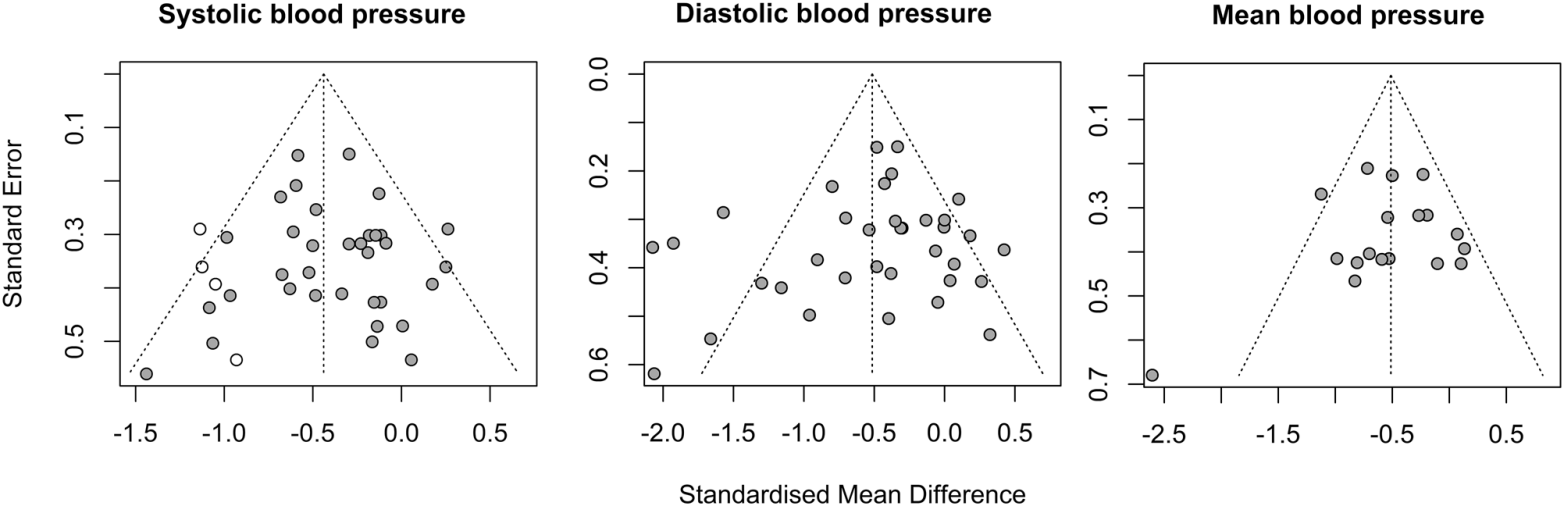
Publication bias representation by trim and fill funnel plots. White circles represent possible omitted studies.

In general, studies present a low to moderate risk of bias in all domains (Fig. 5). Just one study mentions the previous existence of protocols or clinical study records, making it difficult to analyse bias related to the selection of reported results. None of the studies reported intention-to-treat analysis, conflicts of interest or participants were blinded to interventions, what is expected in physical exercise interventions and does not seem to be a major problem in this type of intervention^59^. Tests for subgroup differences showed no differences between studies at high risk of bias in relation to others in SBP (*p* = 0.37) and MBP (*p* = 0.11). A difference was identified in DBP (*p* < 0.01), however the effect favors studies with lower risk of bias (SMD = − 0.58 [− 0.77; − 0.38]) compared to studies with high risk of bias (SMD = 0.03 [− 0.35; 0.41]). The publication bias tests showed no asymmetries in the funnel plot for SBP (Egger’s regression *p* = 0.818), DBP (Egger’s regression *p* = 0.398) or MBP reactivity (Egger’s regression *p* = 0.557). However, four omitted results are expected by trim and fill funnel plots only in SBP (Fig. 6). Quality of evidence analysis show moderate (SBP) to high (DBP) certainty of evidence (Table 3).

**Table 3:** Quality of evidence by Grading of Recommendations Assessment , Development and Evaluation (GRADE).

Outcome	No of studies (n)	Study design	Risk of bias	Inconsistency	Indirectness	Imprecision	Other considerations	Effect SMD (95% CI)	Certainty
Systolic blood presure	23 (589)	Randomised trials	Not serious	Not serious	Not serious	Not serious	Publication bias strongly suspected^a^	− **0.38** (− 0.49 to − 0.27)	⨁⨁⨁◯ Moderate
Diastolic blood presure	24 (619)	Randomised trials	Not serious	Not serious	Not serious	Not serious	None	− **0.51** (− 0.7 to − 0.33)	⨁⨁⨁⨁ High

n, number of participants; CI, confidence interval; SMD, standardised mean difference.

^a^Four omitted results are expected by trim and fill funnel plots.

Significant values are in [bold].

## Discussion

Our main results were that 60% (18 out of 30) of the included studies reported attenuated BP peaks (either in SBP, DPB, and/or MBP) after acute exercise and none showed deleterious results from the exercise. The metanalytic results suggest that acute exercise attenuates BP reactivity to stress. This effect occurred mutually in SBP (SMD = − 0.38 [− 0.49; − 0.27]), DBP (SMD = − 0.51 [− 0.70; − 0.33]) and MBP (SMD = − 0.51 [− 0.72; − 0.31]) in magnitudes similar to previous meta-analyse about the effects of acute aerobic exercise (SBP Effect size = 0.38; DBP Effect size = 0.40)^12^. Besides that, only 30% of the studies included non-aerobic exercises which make the results for these exercise mode difficult to generalize. Lastly, there is a scarcity of studies with hypertensive individuals (10%) and with a population over 40 years old (13%). As for the quality of the evidence, the possible publication bias in SBP may be due only to a physiological response, since the expected omitted results would favor interventions with exercises. In this way, the quality of evidence of SBP would be high.

In this sense, we reaffirm the need for studies with high cardiovascular risk patients, since these responses contribute to the construction of the clinical picture of these patients and may indicate an increase in left ventricular mass^9^, augmented carotid atherosclerosis^60^, increased risk of cardiovascular mortality^61^, development of hypertension^11^, and an increased risk of developing several cardiovascular diseases^2,4^. We also extend this need for studies with the elderly, who, in addition to having the aforementioned advantages of having a high incidence of cardiovascular diseases^62^, seem to have very promising responses when compared to younger people^63^, so studies exploring specific age stratus are needed. We also emphasize that, in addition to expanding and confirming favorable responses to aerobic exercise^12^, the present study is, as far as we know, the first to demonstrate positive meta-analytic effects of resistance exercise in BP reactivity. It is worth mentioning that these results are anchored in a smaller volume of evidence, and should be interpreted with caution, but it provides an optimistic direction for future studies with this exercise mode.

Regarding intervention characteristics, studies that compare different exercise loads showed mixed results. As an example, three studies evaluated different exercise intensities, and one was favorable to higher intensities^25^, another obtained a very discreet advantage at greater intensities^47^, and the latter found no differences between groups^40^. Concerning exercise session duration, a study shows favorable effects of longer sessions^51^, and the others found no differences^32,46^. Finally, a study compared continuous aerobic exercise of moderate intensity with high-intensity interval exercise and also found no significant differences^50^. Although still scarce, the results with resistance exercises are also inconsistent, with higher training volumes (50 min compared to 30 min) seem to be more effective at moderate intensity^51^, but low^41^ or very high intensities^42^ show little or no favorable results when performed for 30–45 min. Thus, evidence on differences arising from the characteristics of exercise load control is still scarce, therefore a meta-analysis clustering intensity groups was not possible. However, the evidence is greater in moderate exercises for 30–60 min.

Overall, when exploring studies heterogeneity, we found that reductions in peak DBP appear to be more heterogeneous than those in SBP. In addition, the greatest effects found are usually in subgroups with fewer studies, and most of the heterogeneity seems to be driven by studies published before the year 2000. So, regarding the effect on DBP response, several results must be highlighted. The first is that, in sex comparisons, the high heterogeneity in men (I^2^ = 74%) draws attention and seems to be explained by Ebbesen et. al study^32^. This study has a very favorable effect on exercise and is not overlapping with other studies, and with its suppression, we have important reductions in heterogeneity and effect size (I^2^ = 25%; SMD = − 0.33[− 0.54; − 0.12]). The large volume of exercise in this study (from 60 to 120 min) may also explain this difference. Also, there is an important DBP responses heterogeneity related to studies that include both sexes (I2 = 76%), which is expected due to the lower specificity of the population. A point that still draws a lot of attention in comparisons by sex, is the large effect size related to studies without defined sex (− 1.16 [− 1.72; − 0.59]). However, this subgroup has only 2 studies, and one of them^31^ has an exceptionally large effect (− 2.06 [− 3.28; − 0.85]).

Besides that, there is high DBP responses heterogeneity in studies with aerobic exercise (I^2^ = 73%). The main characteristics of these studies that may explain their differences to the others in the subgroup are the inclusion of hypertensive patients^31,39^, the high volume of exercise (from 60 to 120 min)^32^ and the self-selected exercise intensity strategy^34^. Regarding the studies with resistance exercises, the heterogeneity is significantly reduced (from I^2^ = 60%, to I^2^ = 22%, with SMD = − 0.72[− 1.00; − 0.45]) with the suppression of one study^42^. This heterogeneity might be explained by the alternative training with an intensity much higher than that of other studies (eccentric phase training at 120% of 1 repetition maximum test). Furthermore, the high DBP responses heterogeneity (I^2^ = 88%) and the greater effect size (− 0.96 [− 1.69; − 0.22]) in studies with RCT design are also noteworthy. In this regard, when we remove the study from Ebbesen et al.^32^, drastically reduces the heterogeneity and effect size of the subgroup (I^2^ = 0%; SMD = − 0.28[− 0.64; 0.08]). This might be explained by the large volume of exercise in this study (from 60 to 120 min) compared to the others in the subgroup (up to 45 min).

Considering types of stressors, there are moderate effects in studies that present physical tests (isolated or both), but mental tests alone have small effects. This may indicate greater effects of exercise in situations of physical stress than in situations of mental stress. Furthermore, the high DBP responses heterogeneity of the group with associated physical and mental stressors (I^2^ = 86%) draws attention, but it was expected due to the heterogeneity of the stress tests. However, 2 studies stand out in this subgroup for having lower results that don’t overlap with the others^32,39^. The main characteristics of these studies that can explain their differences in relation to the others are the large volume of training (from 60 to 120 min) in one study^32^ and the inclusion of hypertensive patients in the other^39^. Another interesting fact is that both studies were carried out in the 1990s. However, it is difficult to be precise about the role of the stressors types, since most of the tests inflict isolated mental stress, and after the suppressions proposed in this analysis, only 4 studies remained in the subgroup with both types of stressors. The suppression of these studies reduces the associated stressor types subgroup heterogeneity (I^2^ = 0%) and effect size (0.02 [− 0.37; 0.41]), generating a situation in which studies with isolated effects (mental or physical) have effects weak to moderate while in tests with both associated have null effects. These studies also have no overlap with the others from the multiple stressors subgroup of DBP analysis, and their suppression generates a reduction in heterogeneity (from I^2^ = 85%, to I^2^ = 0%) and in effect size (from − 0.68 [− 1.24; − 0.13], to − 0.17 [− 0.45; 0.11]) in this subgroup. However, even if the effect on these two subgroups becomes null, we emphasize that they are small subgroups (between 4 and 5 studies after suppressions) and the overall effect remains favorable to exercise (SMD = − 0.37 [− 0.51; − 0.23]).

Another source of heterogeneity could be the fact that several stress tests were used, from classically standardized and widely used protocols such as the cold pressor test^64^ to less restricted but with greater ecological validity as study task^26^. In this sense, we believe that a convergence of these characteristics is necessary, to combine sufficient standardization of methods with greater continuity with the stress experienced in daily life^5^. Thus, studies with multiple stressors such as the Trier Social Stress Test (that includes public speaking with a simulated job interview and arithmetic task) and the Maastricht Acute Stress Test (that includes cold pressure stress, negative feedback, and arithmetic task) seem to be good alternatives for future studies^5^.

As the types of stressors, their mechanisms of action are also diverse. So, these stressor types differences may have occurred due to different mechanisms triggers, with mental stressors appearing to activate frontal lobes and limbic structures that connect to the hypothalamus, while physical stressors recruit the brainstem and hypothalamus^1,5^. Furthermore, in the present study, physical stressors are mainly represented by the cold pressor test, and in this sense, local exposure to cold causes a rapid vasoconstriction response as a thermoregulatory measure^65^. This response is primarily mediated by noradrenaline via the α2-adrenergic receptor, and subsequently by peripheric responses, such as reduction of endothelial nitric oxide synthase activity (reducing the nitric oxide-mediated vasodilation) and increase in mitochondrial reactive oxygen species (resulting in vasoconstriction via Rho-kinase signaling mechanisms)^65^. But we emphasize that the mechanisms responsible for the responses to different stress tests are still poorly explored in the literature and should be encouraged.

Furthermore, in a broader sense, when a stressful situation is imposed, it generates a response that includes diverse mechanisms^1–3^. So, it is an instantaneous activation of the autonomic nervous system to produce physiological arousal with parasympathetic withdrawal^66,67^, changing the dynamics of neural networks, with a dominance of salience network over executive control and default mode networks^68,69^, and stimulation of the hypothalamic–pituitary–adrenal axis^1,2^. These central changes generate increased release of catecholamines^3,70^, opioids/β endorphin^71,72^, and specially cortisol^73,74^. So, the isolated and interaction effects^75^ of these mechanisms may explain the BP reactivity to stress^3,76^. Exercise, in turn, seems to mitigate stress reactivity by reducing vascular resistance^39^, norepinephrine^77^, and hypothalamic–pituitary–adrenal axis responses^78^, in addition to causing increased β2-mediated vasodilation^77^ and levels of endorphins^79^. Finally, there are also psychosocial effects of exercise such as improved self-efficacy and distraction from negative feelings^80^.

It should be emphasized that the present review has some limitations, such as the multiplicity of stress tests and exercise prescription, which makes it difficult to generalize the results. Besides that, these results are mostly in healthy and young populations and therefore cannot be easily generalized to populations with different health conditions. Thus, in future studies, we encourage the research of stressors similar to everyday life, involving different situations, sensations, emotions, and especially extended stressors like those found in sports, social fragility, and scholar/work environment. In this sense, we highlight a study^26^, which despite achieving null results, has an interesting approach with great ecological validity (40 min of studying with undergraduate students). Finally, we also encourage studies that allow a better understanding of exercise load control (e.g., intensity, volume), and in older populations with different morbidities, which can help to improve individual intervention strategies. As a clinical application, physical exercise can be a strategy to reduce hypertensive peaks in individuals who present stressful situations during activities of daily living, thereby reducing cardiovascular risk.

## Conclusion

In summary, acute physical exercise lowers SBP, DBP, and MBP reactivity to stressor tests. However, these results refer mainly to healthy younger adults, who represented a largest part of the analyzed sample. Moreover, given the small magnitude of effects, the clinical relevance of this result must be interpreted with caution and be better explored. Further studies would help understand the effect of different exercise modalities to apply to different clinical profiles (e.g. normotensive vs. hypertensive subjects), helping explore the clinical application of this screening tool. Also, in future studies, we encourage the researcher to use stressors similar to everyday life, making research results more applicable.

## Supplementary Information

Supplementary Information.

## Data Availability

The datasets generated during and/or analysed during the current study are available from the corresponding author on reasonable request.
