# Supplementary material for: A single session of exercise reduces blood pressure reactivity to stress: a systematic review and meta-analysis
Source: Sci Rep. 2022 Jul 12;12:11837. doi: 10.1038/s41598-022-15786-3 (PMC9276760; doi:10.1038/s41598-022-15786-3)
Supplement: Supplementary file 1 — Supplementary Information. [file 41598_2022_15786_MOESM1_ESM.docx]

**Excluded studies**

**No blood pressure under stress**

Pokhrel, B. R., Malik, S. L., Ansari, A. H., Paudel, B. H., Sinha, R., Sinha, M., B.R., P., S.L., M., A.H., A., B.H., P., R., S., M., S., Pokhrel, B. R., Malik, S. L., Ansari, A. H., Paudel, B. H., Sinha, R., & Sinha, M. (2013). Effect of sub-maximal exercise stress on cold pressor pain: A gender based study. Kathmandu University Medical Journal, 11(41), 54–59. https://doi.org/10.3126/kumj.v11i1.11028

Rocha, N. G., Sales, A. R. K., Miranda, R. L., Silva, M. S., Silva, J. F. R., Silva, B. M., Santos, A. A., & Nóbrega, A. C. L. (2015). Aerobic exercise modulation of mental stress-induced responses in cultured endothelial progenitor cells from healthy and metabolic syndrome subjects. Life Sciences, 123, 93–99. https://doi.org/10.1016/j.lfs.2014.12.026

Taylor, A., & Katomeri, M. (2006). Effects of a brisk walk on blood pressure responses to the Stroop, a speech task and a smoking cue among temporarily abstinent smokers. Psychopharmacology, 184(2), 247–253. https://doi.org/10.1007/s00213-005-0275-1

D., K., & R., C.-D. (2012). Effect of a cold pressor test (CPT) on the cardiovascular (CV) responses to static handgrip exercise and peak force production. FASEB Journal, 26. http://www.embase.com/search/results?subaction=viewrecord&from=export&id=L70856469

F.S., R., J.A., M.-D., M., M., L., B., & T., C. (2015). The effect of exercise and distraction on blood pressure recovery following an anger-provoking stressor in normotensive young adults. Journal of Psychophysiology, 29(2), 45–54. https://doi.org/10.1027/0269-8803/a000133

Green, S., & Cameron, E. (2015). Interactive effect of acute sympathetic activation and exercise intensity on the dynamic response characteristics of vascular conductance in the human calf muscle. European Journal of Applied Physiology, 115(5), 879–890. https://doi.org/10.1007/s00421-014-3069-5

K., W., R., W., N., K., B., V. D., J., S., & R., F. (2017). The influence of habitual and acute exercise on SNS and HPA axis responses to psychosocial stress-A randomized, controlled trial. Psychoneuroendocrinology, 83, 77. https://doi.org/10.1016/j.psyneuen.2017.07.445

M., S., S., S., & A., S. (2019). Effect of acute bout of moderate-intensity physical exercise on parameters of stress and cognitive functions. National Journal of Physiology, Pharmacy and Pharmacology, 9(11), 1068–1072. https://doi.org/10.5455/njppp.2019.9.0828014082019

Reims, H. M., Sevre, K., Fossum, E., Mellem, H., Eide, I. K., & Kjeldsen, S. E. (2005). Adrenaline during mental stress in relation to fitness, metabolic risk factors and cardiovascular responses in young men. Blood Pressure, 14(4), 217–226. https://doi.org/10.1080/08037050510034275

Szijgyarto, I. C., King, T. J., Ku, J., Poitras, V. J., Gurd, B. J., & Pyke, K. E. (2013). The impact of acute mental stress on brachial artery flow-mediated dilation differs when shear stress is elevated by reactive hyperemia versus handgrip exercise. Applied Physiology, Nutrition, and Metabolism = Physiologie Appliquee, Nutrition et Metabolisme, 38(5), 498–506. https://doi.org/10.1139/apnm-2012-0328

Taylor, A. H., & Oliver, A. J. (2009). Acute effects of brisk walking on urges to eat chocolate, affect, and responses to a stressor and chocolate cue. An experimental study. Appetite, 52(1), 155–160. https://doi.org/10.1016/j.appet.2008.09.004

Wooller, J. J., Rogerson, M., Barton, J., Micklewright, D., & Gladwell, V. (2018). Can Simulated Green Exercise Improve Recovery From Acute Mental Stress? Frontiers in Psychology, 9, 2167.

Yamagata, T., & Sako, T. (2020). High cardiovascular reactivity and muscle strength attenuate hypotensive effects of isometric handgrip training in young women: A randomized controlled trial. Clinica

P.D., D. (1992). Isometric exercise increases the blood pressure response to psychological stress. Journal of Psychophysiology, 6(1), 11–16. http://www.embase.com/search/results?subaction=viewrecord&from=export&id=L22212709

Dora, K., Suga, T., Tomoo, K., Sugimoto, T., Mok, E., Tsukamoto, H., Takada, S., Hashimoto, T., & Isaka, T. (2021). Similar improvements in cognitive inhibitory control following low-intensity resistance exercise with slow movement and tonic force generation and high-intensity resistance exercise in healthy young adults: a preliminary study. The Journal of Physiological Sciences : JPS, 71(1), 22. https://doi.org/10.1186/s12576-021-00806-0

Lindgren, M., Alex, C., Shapiro, P. A., McKinley, P. S., Brondolo, E. N., Myers, M. M., Choi, C. J., Lopez-Pintado, S., & Sloan, R. P. (2013). Effects of aerobic conditioning on cardiovascular sympathetic response to and recovery from challenge. Psychophysiology, 50(10), 963–973. https://doi.org/10.1111/psyp.12078

**Other study design**

Brownley, K. A., Hinderliter, A. L., West, S. G., Girdler, S. S., Sherwood, A., & Light, K. C. (2003). Sympathoadrenergic mechanisms in reduced hemodynamic stress responses after exercise. Medicine and Science in Sports and Exercise, 35(6), 978–986. https://doi.org/10.1249/01.MSS.0000069335.12756.1B

Albright, G. L., Andreassi, J. L., & Steiner, S. S. (1988). Interactive effects of type A personality and psychological and physical stressors on human cardiovascular functions. International Journal of Psychophysiology : Official Journal of the International Organization of Psychophysiology, 6(4), 315–326. https://doi.org/10.1016/0167-8760(88)90019-0

E.A., G., M.M., P., Y., Z., M.C., O., J.R., S., & B.D., J. (2018). Acute aerobic exercise does not alter the pressor response to the cold pressor test. FASEB Journal, 32(1). http://www.embase.com/search/results?subaction=viewrecord&from=export&id=L622545768

F., M., S., K., & R.G., K. (2015). Influence of aerobic cycling on stress test-related arterial compliance and its relationship to maximal oxygen consumption. Journal of Hypertension, 33, e275–e276. https://doi.org/10.1097/01.hjh.0000468204.08519.ed

Medeiros, R. F., Silva, B. M., Neves, F. J., Rocha, N. G., Sales, A. R. K., & Nobrega, A. C. (2011). Impaired hemodynamic response to mental stress in subjects with prehypertension is improved after a single bout of maximal dynamic exercise. Clinics (Sao Paulo, Brazil), 66(9), 1523–1529. https://doi.org/10.1590/s1807-59322011000900003

N.G.R., R., R.M.F., F., A.R.K.S., S., F.S.P., P., T.C.B., B., T.M.S., S., F.T.C., C., B.M.S., S., F.J.N., N., & A., N. (2010). Presence of the 894G{\textgreater}T polymorphism of endothelial nitric oxide synthase modifies the hemodynamic responses to a mental stress challenge performed after dynamic exercise. European Heart Journal, 31, 704. https://doi.org/10.1093/eurheartj/ehq289

S., K., F., M., & R.G., K. (2014). Influence of high-intensity interval training on peripheral and central blood pressure at rest and during a cold pressor test. Journal of the American Society of Hypertension, 8(4), e55. https://doi.org/10.1016/j.jash.2014.03.122

Milatz, F., Ketelhut, S., Ketelhut, S., &#38; Ketelhut, R. G. (2015). Favorable effect of aerobic exercise on arterial pressure and aortic pulse wave velocity during stress testing. VASA. Zeitschrift Fur Gefasskrankheiten(4), 271–276. https://doi.org/10.1024/0301-1526/a000441</div>

Móra, Á., Komka, Z., Végh, J., Farkas, I., Kocsisné, G. S., Bosnyák, E., Szmodis, M., Ligetvári, R., Csöndör, É., Almási, G., Oláh, A., Kemper, H. C. G., Tóth, M., & Ács, P. (2022). Comparison of the Cardiovascular Effects of Extreme Psychological and Physical Stress Tests in Male Soccer Players. International Journal of Environmental Research and Public Health, 19(2). https://doi.org/10.3390/ijerph19020715

G.P., T., J.V., V. Z., A.T., G., & S.E., W. (2019). The effects of acute exercise on psychophysiological responses to stress. Psychosomatic Medicine, 81(4), A85. https://doi.org/10.1097/PSY.0000000000000699

Morissette, M. P., Cordingley, D. M., Duhamel, T. A., & Leiter, J. R. S. (2020). The Effects of Acute Anaerobic Exercise on the Cardiovascular and Metabolic Response to the Cold Pressor Test in Healthy Adult Males. International Journal of Exercise Science, 13(3), 1729–1740.

Vianna, L. C., Silva, B. M., & Nóbrega, A. C. L. (2014). Sex differences in blood pressure responses to mental stress are abolished after a single bout of exercise: underlying hemodynamic mechanisms. The Journal of Physiological Sciences, 64(3), 213–219. https://doi.org/10.1007/s12576-014-0306-y

Chandrakumar, D., Dyck, D., H Boutcher, S., & N Boutcher, Y. (2018). Acute Effect of Aerobic Exercise on Cardiovascular Reactivity of Overweight Males. International Journal of Human Movement and Sports Sciences, 6(3), 47–54. https://doi.org/10.13189/saj.2018.060301

**Studies with children**

A.R., A.-G., G.A., A.-V., A., S.-H., J., M.-J., & M., M.-D. (2019). Exercise protects cardiovascular recovery from stress in a sample of black ethnicity adolescents. Gazzetta Medica Italiana Archivio per Le Scienze Mediche, 178(7–8), 491–500. https://doi.org/10.23736/S0393-3660.18.03889-5

S.B., R., D.A., B. A., F.O., C. O., J.F.V.N., de M., I.R.C., de S., H.G., S., C.S.G., C., Rauber, S. B., Boullosa, D. A., Carvalho, F. O., de Moraes, J. F. V. N., de Sousa, I. R. C., Simões, H. G., Campbell, C. S. G., S.B., R., D.A., B. A., F.O., C. O., J.F.V.N., de M., I.R.C., de S., … Campbell, C. S. G. (2014). Traditional games resulted in post-exercise hypotension and a lower cardiovascular response to the cold pressor test in healthy children. Frontiers in Physiology, 5 JUN, 235. https://doi.org/10.3389/fphys.2014.00235

**No acute exercises intervention**

Brindle, R. C., Whittaker, A. C., Bibbey, A., Carroll, D., & Ginty, A. T. (2017). Exploring the possible mechanisms of blunted cardiac reactivity to acute psychological stress. International Journal of Psychophysiology : Official Journal of the International Organization of Psychophysiology, 113, 1–7. https://doi.org/10.1016/j.ijpsycho.2016.12.011

C.V., B., S.J.E., L., K., M., & A.C., W. (2017). Revisiting the cross-stressor adaptation hypothesis: Effects of ageing and aerobic fitness on stress reactivity. Brain and Neuroscience Advances, 1, 31–32. https://doi.org/10.1177/2398212817705279

Chafin, S., Christenfeld, N., & Gerin, W. (2008). Improving cardiovascular recovery from stress with brief poststress exercise. Health Psychology : Official Journal of the Division of Health Psychology, American Psychological Association, 27(1S), S64-72. https://doi.org/10.1037/0278-6133.27.1(Suppl.).S64

Cheng, L. S., Carmelli, D., Hunt, S. C., & Williams, R. R. (1997). Segregation analysis of cardiovascular reactivity to laboratory stressors. Genetic Epidemiology, 14(1), 35–49. https://doi.org/10.1002/(SICI)1098-2272(1997)14:1<35::AID-GEPI3>3.0.CO;2-4

Currie, K. D., Sless, R. T., Notarius, C. F., Thomas, S. G., Goodman, J. M., K.D., C., R.T., S., C.F., N., S.G., T., & J.M., G. (2017). Absence of resting cardiovascular dysfunction in middle-aged endurance-trained athletes with exaggerated exercise blood pressure responses. Journal of Hypertension, 35(8), 1586–1593. https://doi.org/10.1097/HJH.0000000000001365

Dvivedi, J., Kaur, H., & Dvivedi, S. (2008). Effect of 1 week “61-points relaxation training” on cold pressor test induced stress in premenstrual syndrome. Indian Journal of Physiology and Pharmacology, 52(3), 262–266.

G., S., K.K., M., & L., S. (2007). Shavasana-Relaxation technique to combat stress. Journal of Bodywork and Movement Therapies, 11(2), 173–180. https://doi.org/10.1016/j.jbmt.2007.01.002

Kalfon, R., Campbell, J., Alvarez-Alvarado, S., & Figueroa, A. (2015). Aortic Hemodynamics and Arterial Stiffness Responses to Muscle Metaboreflex Activation With Concurrent Cold Pressor Test. American Journal of Hypertension, 28(11), 1332–1338. https://doi.org/10.1093/ajh/hpv043

Koch, D. W., Leuenberger, U. A., & Proctor, D. N. (2003). Augmented leg vasoconstriction in dynamically exercising older men during acute sympathetic stimulation. The Journal of Physiology, 551(Pt 1), 337–344. https://doi.org/10.1113/jphysiol.2003.042747

Maekawa, K., Kuboki, T., Yamashita, A., & Clark, G. T. (1999). Effect of cold pressor stimulation (4 degrees C) on human masseter muscle haemodynamics during and after sustained isometric contraction. Archives of Oral Biology, 44(11), 969–973. https://doi.org/10.1016/s0003-9969(99)00093-x

P.M., M.-L., A.L., T., D., A., M.C., S., D.R., R., P.S., R., & L.C., V. (2017). Impact of metaboreflex activation on cerebrovascular reactivity index in healthy young men. FASEB Journal, 31(1). http://www.embase.com/search/results?subaction=viewrecord&from=export&id=L616910692

R.M., L., R., G., A.B., G., S.G.R., N., F.L., P., & L.C., V. (2015). Blood pressure reactivity to mental stress is attenuated following resistance exercises in treated older hypertensive women. Autonomic Neuroscience: Basic and Clinical, 192, 111. https://doi.org/10.1016/j.autneu.2015.07.182

Stuckless, T. J. R., & Pyke, K. E. (2015). The impact of a cold pressor test on brachial artery handgrip exercise-induced flow-mediated dilation. Vascular Medicine (London, England), 20(5), 409–416. https://doi.org/10.1177/1358863X15586473

Wasmund, W. L., Westerholm, E. C., Watenpaugh, D. E., Wasmund, S. L., & Smith, M. L. (2002). Interactive effects of mental and physical stress on cardiovascular control. Journal of Applied Physiology

Kelleher, C., Ferriss, J. B., Ross, H., & O’Sullivan, D. J. (1987). The Pressor Response to Exercise and Stress in Uncomplicated Insulin-Dependent Diabetes. J Hum Hypertens, 1(1), 59–64.

**No control without exercise**

Gauche, R., Lima, R. M., Myers, J., Gadelha, A. B., Neri, S. G. R., Forjaz, C. L. M., & Vianna, L. C. (2017). Blood pressure reactivity to mental stress is attenuated following resistance exercise in older hypertensive women. Clinical Interventions in Aging, 12, 793–803. https://doi.org/10.2147/CIA.S130787

Ketelhut, S., Milatz, F., Heise, W., & Ketelhut, R. G. (2016). Influence of a high-intensity interval training session on peripheral and central blood pressure at rest and during stress testing in healthy individuals. VASA. Zeitschrift Fur Gefasskrankheiten, 45(5), 373–377. https://doi.org/10.1024/0301-1526/a000560

Rashed, H. M., Leventhal, G., Madu, E. C., Reddy, R., & Cardoso, S. (1997). Reproducibility of exercise-induced modulation of cardiovascular responses to cold stress. Clinical Autonomic Research : Official Journal of the Clinical Autonomic Research Society, 7(2), 93–96. https://doi.org/10.1007/BF02267753

Steptoe, A., Kearsley, N., & Walters, N. (1993). Cardiovascular activity during mental stress following vigorous exercise in sportsmen and inactive men. Psychophysiology, 30(3), 245–252. https://doi.org/10.1111/j.1469-8986.1993.tb03350.x

Azevêdo, L. M., de Souza, A. C., Santos, L. E. S., Miguel Dos Santos, R., de Fernandes, M. O. M., Almeida, J. A., & Pardono, E. (2017). Fractionated Concurrent Exercise throughout the Day Does Not Promote Acute Blood Pressure Benefits in Hypertensive Middle-aged Women. Frontiers in Cardiovascular Medicine, 4, 6. https://doi.org/10.3389/fcvm.2017.00006

LaManca, J. J., Peckerman, A., Sisto, S. A., DeLuca, J., Cook, S., & Natelson, B. H. (2001). Cardiovascular responses of women with chronic fatigue syndrome to stressful cognitive testing before and after strenuous exercise. Psychosomatic Medicine, 63(5), 756–764. https://doi.org/10.1097/00006842-200109000-00009

N.H., P., W.A., S., & S.K., S. (2013). Short-term effects of isotonic handgrip exercise on cardiovascular autonomic functions in healthy young adolescents. Indian Journal of Physiology and Pharmacology, 57(5), 185. http://www.embase.com/search/results?subaction=viewrecord&from=export&id=L71857327

Szijgyarto, I. C., Poitras, V. J., Gurd, B. J., & Pyke, K. E. (2014). Acute psychological and physical stress transiently enhances brachial artery flow-mediated dilation stimulated by exercise-induced increases in shear stress. Applied Physiology, Nutrition, and Metabolism = Physiologie Appliquee, Nutrition et Metabolisme, 39(8), 927–936. https://doi.org/10.1139/apnm-2013-0384

Brownley, K. A., Hinderliter, A. L., West, S. G., Girdler, S. S., Sherwood, A., & Light, K. C. (2003). Sympathoadrenergic mechanisms in reduced hemodynamic stress responses after exercise. Medicine and Science in Sports and Exercise, 35(6), 978–986. https://doi.org/10.1249/01.MSS.0000069335.12756.1B

Ketelhut, S., Ketelhut, R. G., Kircher, E., Röglin, L., Hottenrott, K., Martin-Niedecken, A. L., & Ketelhut, K. (2022). Gaming Instead of Training? Exergaming Induces High-Intensity Exercise Stimulus and Reduces Cardiovascular Reactivity to Cold Pressor Test. Frontiers in Cardiovascular Medicine, 9, 798149. https://doi.org/10.3389/fcvm.2022.798149

Rocha, N., Neves, F., Silva, B., Sales, A., & Nóbrega, A. (2012). The 894G>T endothelial nitric oxide synthase genetic polymorphism affects hemodynamic responses to mental stress performed before and after exercise. European Journal of Applied Physiology, 112(3), 877–886. http://search.ebscohost.com/login.aspx?direct=true&db=s3h&AN=71509341&amp

Milatz, F., Ketelhut, S., & Ketelhut, R. G. (2017). Acute Effects of Moderate Continuous Training on Stress Test-Related Pulse Pressure and Wave Reflection in Healthy Men. High Blood Pressure & Cardiovascular Prevention, 24(1), 61–67. https://doi.org/10.1007/s40292-017-0180-9

Moya-Albiol, L., Salvador, A., Costa, R., Martinez-Sanchis, S., Gonzalez-Bono, E., Ricarte, J., & Arnedo, M. (2001). Psychophysiological responses to the Stroop Task after a maximal cycle ergometry in elite sportsmen and physically active subjects. International Journal of Psychophysiology, 40(1), 47–59. http://search.ebscohost.com/login.aspx?direct=true&db=s3h&AN=SPHS-672054&amp

**Other languages**

A., D. M., C., B., G., P., & B., P. (1991). Blood pressure recordings during a Ruffier exercise test and cycle ergometry. Science and Sports, 6(2), 85–90. http://www.embase.com/search/results?subaction=viewrecord&from=export&id=L21239060

F., M., S., K., & R.G., K. (2014). Influence of acute endurance exercise on peripheral and central blood pressure at rest and during a cold pressor stress test. Perfusion (Germany), 27(2), 56–60. http://www.embase.com/search/results?subaction=viewrecord&from=export&id=L607352230

**Food cointervention**

Faulk, K. E., & Bartholomew, J. B. (2012). The moderating effect of physical activity on cardiovascular reactivity following single fat feedings. Psychophysiology, 49(1), 145–149. https://doi.org/10.1111/j.1469-8986.2011.01283.x
